# Mass testing and treatment for malaria followed by weekly fever screening, testing and treatment in Northern Senegal: feasibility, cost and impact

**DOI:** 10.1186/s12936-020-03313-6

**Published:** 2020-07-14

**Authors:** Ruben O. Conner, Yakou Dieye, Michael Hainsworth, Adama Tall, Badara Cissé, Farba Faye, Mame Demba Sy, Amadou Ba, Doudou Sene, Souleymane Ba, Elhadji Doucouré, Tidiane Thiam, Moussa Diop, Kammerle Schneider, Moustapha Cissé, Mady Ba, Duncan Earle, Philippe Guinot, Richard W. Steketee, Caterina Guinovart

**Affiliations:** 1 PATH Malaria Control and Elimination Partnership in Africa (MACEPA), 2201 Westlake Avenue, Suite 200, Seattle, WA 98121 USA; 2 Programme National de Lutte contre le Paludisme (PNLP), Ministère de la Santé et l’Action Sociale, Dakar, Senegal; 3 Barcelona Institute for Global Health (ISGlobal), Hospital Clínic–Universitat de Barcelona, Rosselló 132, 08036 Barcelona, Spain

**Keywords:** Population-wide interventions, Testing and treatment, *Plasmodium falciparum*, Malaria elimination

## Abstract

**Background:**

Population-wide interventions using malaria testing and treatment might decrease the reservoir of *Plasmodium falciparum* infection and accelerate towards elimination. Questions remain about their effectiveness and evidence from different transmission settings is needed.

**Methods:**

A pilot quasi-experimental study to evaluate a package of population-wide test and treat interventions was conducted in six health facility catchment areas (HFCA) in the districts of Kanel, Linguère, and Ranérou (Senegal). Seven adjacent HFCAs were selected as comparison. Villages within the intervention HFCAs were stratified according to the 2013 incidences of passively detected malaria cases, and those with an incidence ≥ 15 cases/1000/year were targeted for a mass test and treat (MTAT) in September 2014. All households were visited, all consenting individuals were tested with a rapid diagnostic test (RDT), and, if positive, treated with dihydroartemisinin-piperaquine. This was followed by weekly screening, testing and treatment of fever cases (PECADOM++) until the end of the transmission season in January 2015. Villages with lower incidence received only PECADOM++ or case investigation. To evaluate the impact of the interventions over that transmission season, the incidence of passively detected, RDT-confirmed malaria cases was compared between the intervention and comparison groups with a difference-in-difference analysis using negative binomial regression with random effects on HFCA.

**Results:**

During MTAT, 89% (2225/2503) of households were visited and 86% (18,992/22,170) of individuals were tested, for a combined 77% effective coverage. Among those tested, 291 (1.5%) were RDT positive (range 0–10.8 by village), of whom 82% were < 20 years old and 70% were afebrile. During the PECADOM++ 40,002 visits were conducted to find 2784 individuals reporting fever, with an RDT positivity of 6.5% (170/2612). The combination of interventions resulted in an estimated 38% larger decrease in malaria case incidence in the intervention compared to the comparison group (adjusted incidence risk ratio = 0.62, 95% CI 0.45–0.84, p = 0.002). The cost of the MTAT was $14.3 per person.

**Conclusions:**

It was operationally feasible to conduct MTAT and PECADOM++ with high coverage, although PECADOM++ was not an efficient strategy to complement MTAT. The modest impact of the intervention package suggests a need for alternative or complementary strategies.

## Background

With an increasing international focus on malaria elimination, there is a need for interventions that are effective at reducing transmission in different settings [1]. Although there are a few examples of successful elimination efforts [2], questions remain about the effectiveness of specific interventions, the best mix of interventions to be implemented and the operational challenges of scale-up, cost, and sustainability.

Senegal has greatly reduced its malaria burden in the last decade through scale up of control tools [3]. In 2008, the national prevalence of *Plasmodium falciparum* infection by microscopy in children under 5 years old was 5.7%, which decreased to 0.4% in 2017. There is a gradient of transmission intensity, with a gradient in the prevalence from 7.3% in the south-east of the country to near zero in parts of the north [4, 5]. Following this success, the *Programme National de Lutte contre le Paludisme* (PNLP—National Malaria Control Programme) shifted its strategy from control to elimination and partnered with stakeholders to conduct implementation research on potential elimination strategies in different transmission settings to inform the national malaria strategic plan.

Population-wide drug administration strategies are being evaluated as tools to decrease the reservoir of infection and accelerate towards elimination, but more data on their impact and feasibility are needed. Mass test and treat (MTAT) has been proposed as one population-wide drug administration intervention for transmission reduction [6]. This approach involves visiting all households within an area, testing all members for malaria using a rapid diagnostic test (RDT), and treating those that are positive with an anti-malarial drug. Compared to mass drug administration (MDA), where the whole population is given an antimalarial drug, the MTAT approach targets treatment to infected individuals. Thus, the advantages of MTAT are that a smaller quantity of drugs is administered in the community, and persons without infection are not exposed to potential adverse effects of anti-malarials. However, the effectiveness of MTAT may be compromised by the limited sensitivity of standard RDTs, meaning that low density infections (~ <200 parasites/μl) are not detected. Moreover, MTAT, unlike MDA, does not provide the population benefit of a mass prophylactic effect. Studies of MTAT effectiveness have had mixed results. One study demonstrated a 53% reduction in parasite prevalence by RDT [7], but others found no change in incidence [8, 9]. In addition, to sustain the impact of population-wide drug administration strategies, programs must implement activities that clear new infections as they occur.

The PATH Malaria Control and Elimination Partnership in Africa (MACEPA), in collaboration with the PNLP, implemented a package of interventions in northern Senegal, including an MTAT followed by a weekly fever screen, test and treat campaign or case investigation. The goal of this work was to evaluate its impact, feasibility and cost and to provide evidence to inform the national malaria elimination strategy.

## Methods

### Study area

**Fig. 1:**
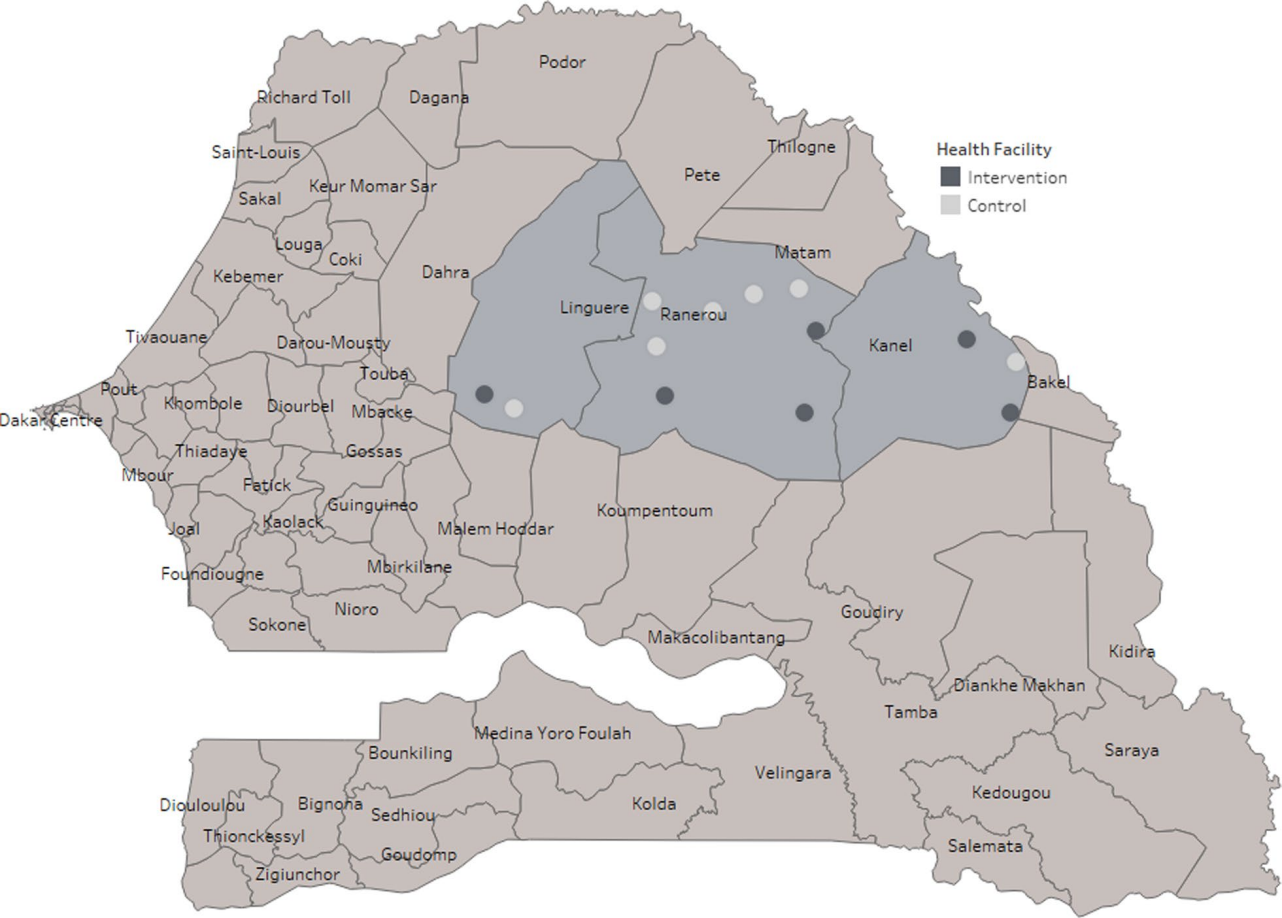
Map of study area

The study was conducted in Kanel, Linguère and Ranérou districts, in Matam and Louga regions in northern Senegal (Fig. 1) from September 2014 to February 2015. The area lies at the edge of the Sahel region and is marked by grasslands, a semi-arid climate and flat topography, with an average population density of about 14 per square kilometre [10]. Malaria transmission is low, with some moderate transmission areas, highly seasonal, with the rainy season occurring once annually between July and January. *Plasmodium falciparum* is responsible for most of the infections and the main malaria vectors are *Anopheles gambiae*, *Anopheles arabiensis*, and *Anopheles funestus* [11, 12]. Malaria control relied on passive case management and bed nets, with no indoor residual spraying being conducted in the area. In the northern region of Senegal, 81% of households reported having at least one insecticide-treated bed net in 2014 [13]. First-line treatment for uncomplicated malaria in the country was either artemether-lumefantrine, artesunate-amodiaquine or dihydro-artemisinin-piperaquine (DHAp) [14].

### Study design

The study used a quasi-experimental design with non-random selection of the intervention and comparison groups. Six health facility catchment areas (HFCA) were purposefully selected by the PNLP to receive the intervention based on the 2013 annual incidence of malaria cases. Seven HFCAs from the same districts were selected as a comparison group for the study. Characteristics of intervention and comparison areas are presented in Additional file 1 showing that average bed net ownership, household size and baseline malaria transmission were lower in comparison areas (Additional file 2, a and b). This is intended as a programmatic evaluation of an intervention that targeted the highest transmission areas within a district, acknowledging the limitations of having to use areas of lower malaria incidence at baseline as the comparison group. The total population in the intervention and comparison areas was approximately 66,000. Within the intervention HFCAs, villages were stratified into different malaria transmission strata based on the 2013 incidence of malaria and were allocated different combinations of interventions as shown in Table 1. Stratum 1 included villages with less than 5 cases per 1000 people per year and only case investigation was conducted from October 2014 to January 2015. Stratum 2 included villages with 5 to 14 cases per 1000 people per year and only weekly mass fever screen, test and treat (referred to as PECADOM++) was implemented during the same period. Stratum 3 included villages with equal or greater than 15 cases per 1000 people per year, where a MTAT was implemented in September 2014, at the beginning of the transmission season, followed by PECADOM++ from October 2014 to January 2015. Given that the intervention areas were small and to avoid a dilution of the impact caused by the surrounding areas not receiving any interventions, villages outside the intervention HFCAs but within a radius of 5 km also received a MTAT. Thus, a larger area was targeted, but the evaluation was confined to the intervention HFCAs, and data from these surrounding villages were not included in the descriptive or impact evaluation analyses. No interventions were implemented in comparison HFCAs, which only received the standard of care provided by the PNLP, which included case management (through health posts, health huts and community health workers) and bed nets, with no seasonal malaria chemoprophylaxis or indoor residual spraying.

**Table 1:** Stratification of intervention villages and implemented interventions

Stratum	Transmission intensity (*P. falciparum* cases per 1000 population per year in 2013)	Catchment population in area	Number of villages	Interventions during high transmission season (October 2014 to January 2015)
1	Very low transmission (< 5 cases/1000/year)	4753	5	Case investigation with FTAT/FSTAT+ focal drug administration for outbreak
2	Low transmission (≥ 5 and < 15 cases/1000/year)	9695	6	PECADOM++
3	Low-moderate transmission (≥ 15 cases/1000/year)	24,925	46	MTAT at the beginning of the 2014 transmission season (September 2014) followed by PECADOM++

FTAT (focal test and treat): all individuals in the index case household were tested with an RDT and treated if positive

FSTAT (focal screen, test and treat): all individuals in the five closest households to the index case household within a 100-m radius were screened for risk factors (reported fever, recent travel or not sleeping under a bed net) and those with at least one were tested with an RDT and treated if positive

PECADOM++: weekly mass fever screen, test and treat along with reactive focal FTAT

### Objectives

The primary objective was to evaluate whether a combination of malaria parasitaemia-clearing strategies could substantially decrease malaria incidence in low transmission areas in Linguère, Ranérou, and Kanel districts. The primary endpoint was the incidence of passively detected, RDT-confirmed *P. falciparum* malaria cases (diagnosed at the health posts or by community health workers) during the 2014 to 2015 transmission season. Secondary objectives were characterizing demographic and spatial patterns of infection to guide the strategy toward elimination; describing the operational feasibility of implementing different parasitaemia-clearing strategies; and estimating the costs of the interventions.

### Study procedures

#### Census

A census was done at the outset of the study and households within the intervention and comparison areas were visited and asked to complete a brief survey including number of individuals in the household, number of sleeping spaces and number of bed nets. Household GPS coordinates were collected as part of this process and were used to locate houses during the interventions. All efforts were made to achieve full coverage and, if households that had been missed during the census were found during the interventions, these were added to the census database.

#### Case investigation with reactive case detection

Case investigation was conducted for all malaria cases (index cases) detected at the health posts or by community health workers in stratum 1 intervention villages. The household of the index case was visited, and FTAT was conducted there. In the five closest neighboring households within a 100-m radius, focal screen test and treat (FSTAT) was conducted: all individuals were screened for risk factors (reported fever in the previous 7 days, recent travel in the previous 2 weeks or not having slept under a bed net the previous night) and those with at least one risk factor were tested with an RDT and treated if positive. Because a high number of secondary cases were found during the FTAT and FSTAT within the same area, these were considered an outbreak. A focal drug administration was conducted as a response in the affected villages: DHAp was administered to all individuals regardless of infection status, using the same inclusion and exclusion criteria described below for the MTAT.

#### PECADOM++

PECADOM stands for “*Prise en charge à domicile*” or case management at the household level, and PECADOM++ was an enhanced PECADOM consisting of weekly mass fever screen, test and treat along with reactive focal test and treat (FTAT). It was implemented in all households in intervention villages in strata 2 and 3 for 16 weeks from October 2014 until January 2015. All households received a weekly visit to screen for reported fever in the previous week. All individuals reporting fever in the previous week received an RDT, and RDT-positive individuals received DHAp. Additionally, in households with a positive RDT, FTAT was done and all individuals received an RDT, and RDT-positive individuals received appropriate treatment.

#### Mass test and treat

All households in stratum 3 intervention villages were targeted for the MTAT. All households were visited by the study team and all individuals older than 2 months of age were invited to participate. Informed consent was obtained from the head of household prior to requesting any information from the household and individual written consent was then obtained from all participating members or from their parents/guardians in the case of children under the age of eighteen. A household questionnaire was then administered to the household head (number of residents, number of sleeping spaces, number of bed nets), and an individual questionnaire was administered to each household member (sociodemographic characteristics, RDT results and treatment, and risk factors for malaria, such as travel history, reported fever, and use of bed net). Information was collected using standardized questionnaires programmed in ODK Collect (an Open Data Kit tool) on smartphones. Three attempts were made to locate individuals who were absent at the time of the survey (either in household where other individuals were present or in empty households).

All consenting individuals were tested for malaria using a Premier Medical First Response^®^ rapid diagnostic test (RDT). Patients who tested positive for malaria were treated with DHAp, unless there were contra-indications. DHAp was chosen based on the longer half-life and prophylactic effect. Since there are contraindications for administering DHAp to women within the first trimester of pregnancy, reproductive age women who were eligible for treatment were asked about their pregnancy status and the timing of their last menstrual period. RDT positive women were not treated and were referred to a clinic if they were within the first trimester of pregnancy or if they were uncertain about their pregnancy status. RDT positive women in the second or third trimester of pregnancy and infants between 2 and 6 months of age were treated with artemether-lumefantrine (AL) instead of DHAp, according to national guidelines. RDT-positive individuals were also referred to a health facility for treatment if they had certain contraindications including allergies to artemisinin drugs, had recently taken artemisinin drugs or other contraindicated drugs, or had previous heart palpitations.

During all three interventions, all cases receiving DHAp were followed up one week later to assess adherence to treatment (reported and through observation of blister) and adverse events through a standardized questionnaire.

#### Data collection and analysis

Field visits were conducted by teams composed of a field worker and a community health worker. Data were entered using handheld Android phones programmed with ODK Collect custom-designed questionnaires. Data was stored on the Android phone and then uploaded daily to a central ODK server. Internal data checks were set up to prevent missing data and identify inconsistent entries. Data analysis was done using Stata 13.1 according to a pre-defined analysis plan.

Risk factor analysis for RDT positivity during the MTAT was conducted using univariable and multivariable logistic regression with RDT result (positive or negative for *P. falciparum*) as the dependent variable. To control for spatial clustering of malaria cases, a fixed effect at the HFCA level and a random effect at the village level were included. Analysis was stratified according to age group: under 10 years of age and 10 years or older, as some of the risk factors in adults (occupation and education) were not relevant for young children. Additionally, separation into two groups allowed for exploration of differences in risk factors for children compared to adults.

To evaluate the impact of the package of interventions on malaria case incidence, a difference-in-differences analysis was done to assess whether the change in the incidence from before and after the intervention was different in comparison versus intervention areas [15], adjusting for baseline differences between the two groups. The model used was a negative binomial regression with weekly malaria cases at the HFCA level as the dependent variable, logged population per HFCA as the offset, a random effect at the HFCA, indicator variables for pre/post and comparison/intervention and an interaction term between pre/post and comparison/intervention. Four HFCA variables which were considered to be a priori confounders available at the HFCA-level were controlled for: average number of bed nets per sleeping space, average number of people per household, average rainfall in millimetres with a two-month lag, and normalized difference vegetation index (NDVI) with a two-month lag. The average number of bed nets per sleeping space and the average number of people per household were calculated using data from the initial census conducted in both the intervention and comparison areas. Both the rainfall and the NDVI were downloaded from the Early Warning and Environmental Monitoring Programme [16] and were averaged by HFCA for the pre and the post-intervention periods. NDVI is a satellite imagery-based measurement which provides a temporally smoothed estimate of the vegetation properties. It is available in 10-day intervals, which were matched to the study period, and the index can take on values in the range of 0 to 200. Weekly malaria case counts were extracted from health facility registers in the intervention and comparison areas, where the completeness and quality of the recording were found to be similar. Cases coming from outside the HFCA were excluded. The evaluation period only included the high malaria transmission season months: Sept 1, 2013, to Jan 31, 2014, for the pre-intervention period and Sept 1, 2014, to Jan 31, 2015, for the post-intervention period. To check the parallel trends assumption of the difference-in-difference analysis, malaria incidence data from the 2012–2013 season was also extracted to assess the trends in the intervention and comparison groups prior to the intervention. A sensitivity analysis was conducted to assess the robustness of the results when including the RDT-positive febrile individuals found during the MTAT and PECADOM++ in the outcome, under the assumption that these individuals would have sought care had the MTAT and PECADOM++ visits not happened.

### Costing

The costs of implementing MTAT, PECADOM++ and case investigation were estimated using an ingredients-based approach [17]. Unit costs and quantity information were collected retrospectively after the completion of fieldwork. The costs were split into three categories: (1) preparation costs, which included the costs of convening meetings with stakeholders prior to the beginning of fieldwork, (2) training costs, which were primarily the cost of salaries, per diems, and supplies for the training sessions conducted in each of the three districts, and (3) implementation costs, which included payment for the community health workers and field workers performing the MTAT intervention, salaries and per diems for technical staff conducting supervision visits, transportation costs, supplies (including RDTs, malaria treatments), and mobile phones for data collection. The summation of these costs provided a total cost for each intervention and these totals were divided by the total population in the targeted areas to estimate the cost per person.

## Results

### Case investigation

Case investigation was conducted from October to December 2014, as there were no cases in January 2015, in stratum 1 villages. Thirteen index cases were passively-detected within stratum 1 intervention villages, and 100% of these were followed up at home. FTAT was conducted in the thirteen index case households, where 97% (198/205) of the individuals were tested, of whom 3.5% (7) had a positive RDT. Forty neighbouring households with 628 individuals were also visited to conduct FSTAT, where 54 (8.6%) had at least one FSTAT risk factor, and 11 (20.4%) had a positive RDT. Seventeen of the 18 secondary cases were found in three villages of the same area, which was considered an outbreak. A focal drug administration was done as a response in twenty households, during which 405 individuals received DHAp on weeks 48 and 49. The mean number of days between index case detection and the start of the household visits was 1.3.

### PECADOM++

PECADOM++ was conducted during 16 weeks from October 2014 to January 2015 in all households in intervention strata 2 and 3 villages. Of the 3577 households registered during the census in the target areas, 93% (3307) were approached for PECADOM++ at least once, with a total of 40,002 visits and a median of 12 visits per household (Additional file 3). Of these, 36,481 visits could be completed (there was someone at home and accepted to participate), of which only 5.2% (1889) (ranging from 2.2% to 10.6% by HFCA) found at least one case of reported fever in the previous week, with a total of 2784 individuals reporting fever. Of these, 2612 (93.8%) were tested with an RDT and 170 (6.5%) were positive. In households with at least one positive RDT, an FTAT was conducted, testing 1271 additional household members, of whom 119 (9.4%) were positive. Thus, the RDT positivity rate of individuals with fever was 6.5%, whereas that of individuals tested in households of RDT positive individuals was 9.4%. Overall, 289 infections were found during 40,002 visits (7.2 infections per 1000 household visits) by 52 teams, with a median of 20 visits/day/team (inter-quartile range 9–39). The coverage, defined as the percentage of households visited per week, varied substantially by HFCA and week, increasing over the course of the study, with a median of 77% (Additional file 4). There was at least one fever case in 40.7% (1295/3179) of households over the whole study period but only 4.8% (154) had at least one positive RDT. Also, 17.3% of infections occurred in households that had other RDT-positives during the study period.

### MTAT

#### Coverage and socio-demographic characteristics

**Fig. 2:**
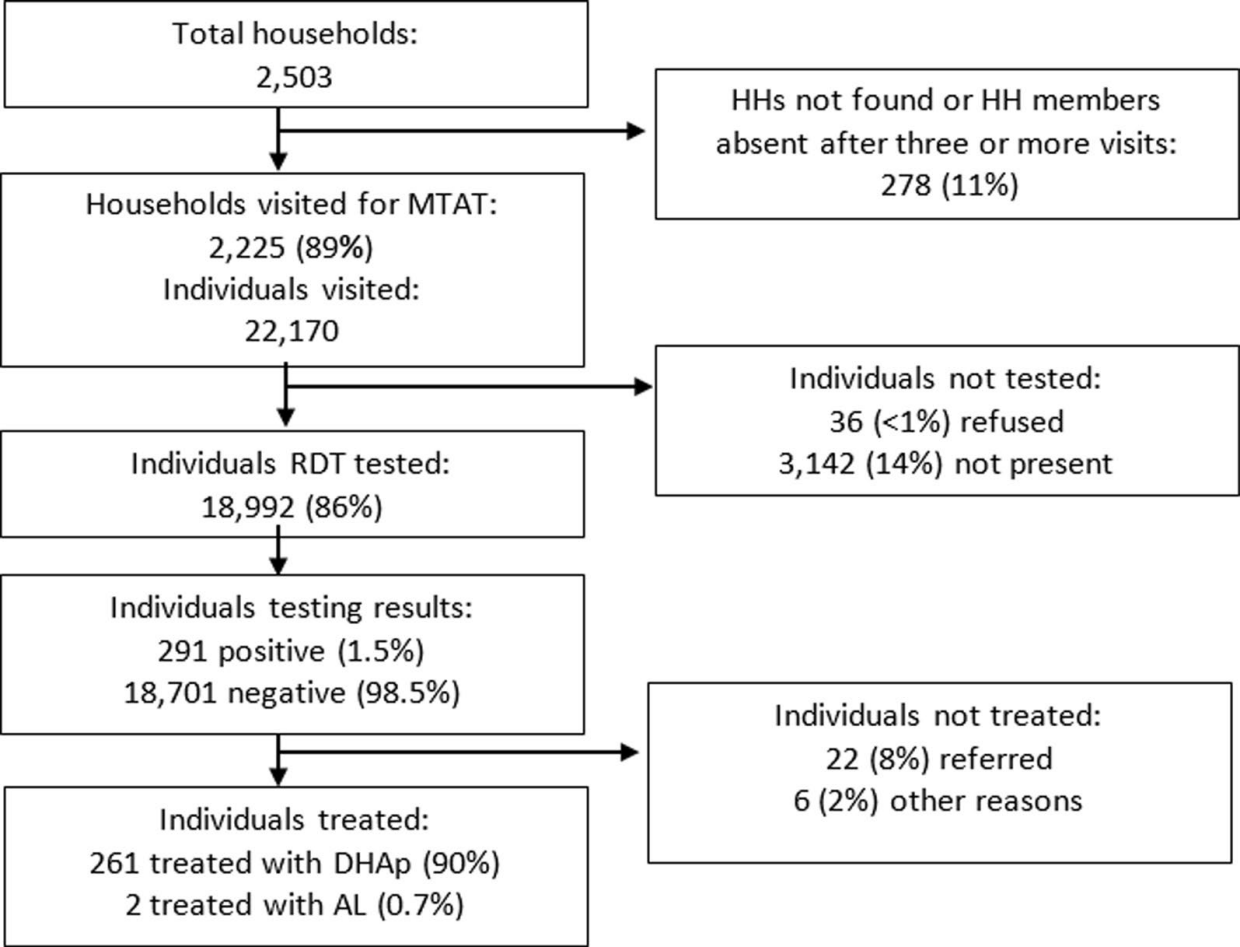
MTAT study profile

The MTAT was conducted in September 2014 in all stratum 3 villages by 80 field teams in 28 days. Of the 2503 households registered during the census in the target areas, the intervention reached 2225, resulting in a household coverage of 89%. Those households included 22,170 individuals, of which 3142 (14%) were not present at the time of the survey and 36 (0.2%) refused. As a result, 18,992 individuals received an RDT, an individual coverage rate of 86%. The combination of household and individual coverage resulted in 77% effective coverage of RDT testing. Each team interviewed and tested 25 individuals per day on average. The study profile is shown in Fig. 2.

Among the population reached, 45% (8639/19,028) of household members were male and 44% (8306/19,025) were younger than ten years of age. Individuals who were absent at the time of the survey were more likely to be male (72% (2271/3142)) and more likely to be over the age of ten (81% (2531/3141)). Among individuals reached, bed net usage was high, with 65% reporting use of a net the previous night. Only 2.6% (495/19,028) of individuals reported travel outside of the district in the previous two weeks, 3.4% (653/19,028) had fever (axillary temperate ≥ 37.5 °C) or reported fever in the previous 24 h and 6.0% (1135/19,028) had fever or reported fever in the previous 7 days.

#### RDT positivity rate and risk factors for RDT positivity

**Fig. 3:**
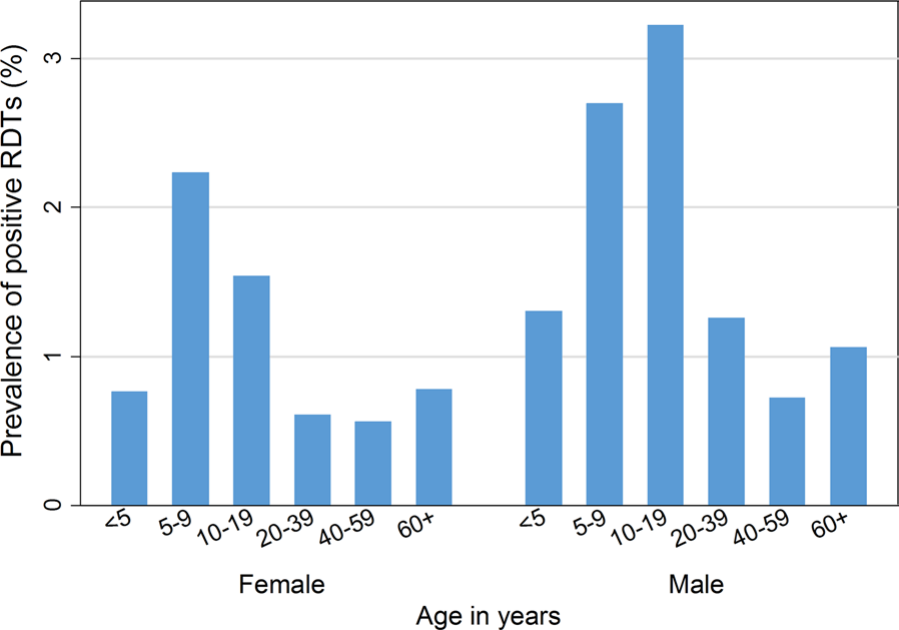
Prevalence of RDT positivity by age group and sex during the MTAT

Of the 18,992 individuals tested as part of the MTAT, 291 were RDT positive, yielding an overall prevalence of 1.5% (range 0%- 10.8% by village). The prevalence of RDT positivity by age group and sex is shown in Fig. 3. Overall, RDT positivity was higher in males and in individuals aged 5–19 years old.

Within the RDT positive group, 261 individuals (90%) were treated with DHAp, 2 (0.7%) were treated with AL, 22 (8%) were referred and 6 (2%) were not treated for other reasons, such as refusal. Among the 208 (80%) individuals that were treated with DHAp and found during the follow up visit, 203 (98%) completed full treatment (according to self-report or observation of the blister pack during the follow-up visit) and 5 (2%) did not complete the course. Among those followed up, 23 (11.1%) had one or multiple adverse events, all of which were mild, and included vomiting (19 cases (9.1%)), fever (8 cases (3.8%)), and itching (1 case (0.5%)).

Among the RDT-positive individuals, 82% (238/291) were younger than 20 years of age, 70% (204/291) were afebrile (no measured fever or reported fever in previous 24 h) and 2% (5/291) had travel history in the previous month. The percentage of those who were afebrile was not significantly different by age group, being 73% (33/45) in children younger than 5 years old, 70% (69/98) in children 5–9 years old, 69% (47/68) in children 10–14 years old, 63% (17/27) in children 15–19 years old and 72% (38/53) in adults aged > 20 years.

Results of the univariable and multivariable logistic regression to assess risk factors for RDT positivity is presented separately for children < 10 years of age (Table 2) and older children and adults (Table 3). In children < 10 years old, the strongest predictors were the presence of more than one RDT-positive individual in the household (OR 21.72, 95% CI 13.83–34.10), fever (OR 13.64, 95% CI 8.18–22.74), and having taken an anti-malarial in the past 3 months (OR 4.11, 95% CI 1.06–15.90). In older children and adults, the strongest predictors were fever (OR 24.75, 95% CI 15.20–40.30), the presence of more than one RDT-positive individual in the household (OR 20.10, 95% CI 12.90–31.31), and having taken an anti-malarial in the past 3 months (OR 8.67, 95% CI 3.30–22.77). Among the older age group, males were twice as likely to test positive for malaria (OR 1.93, 95% CI 1.10–3.36) and education was also significantly associated, with a heightened risk for secondary education (OR 6.12, 95% CI 1.97–19.08). Sleeping under a bed net the previous night or having travelled in the previous 2 weeks were not associated with RDT positivity in either of the two age groups.

**Table 2:** Logistic regression results to assess the risk factors associated with RDT positivity: Individuals < 10 years old

Covariates	N (% of total population < 10 years)	n (distribution of RDT-positive population (%) < 10 years)	Univariable odds ratio (95% CI) p-value	Multivariable adjusted odds ratio (95% CI) p-value
***Sociodemographic characteristics***				
*Sex*				
Female	4136 (50.0%)	60 (43.2%)	1.0 (ref)	1.0 (ref)
Male	4140 (50.0%)	79 (56.8%)	1.32 (0.94–1.85) p = 0.106	1.31 (0.89–1.94) p = 0.175
*Age*				
< 5 years	4304 (52.0%)	45 (32.4%)	1.0 (ref)	1.0 (ref)
5–9 years	3972 (48.0%)	94 (67.6%)	2.29 (1.60–3.28) p < 0.001	2.64 (1.75–3.99) p < 0.001
***Individual malaria risk factors***				
*Used a bed net on previous night*				
No	2749 (33.2%)	41(29.5%)	1.0 (ref)	1.0 (ref)
Yes	5527 (66.8%)	98 (70.5%)	1.19 (0.83–1.72) p = 0.348	1.01 (0.64–1.60) p = 0.961
*Travelled outside of district in previous two weeks*				
No	8133 (98.3%)	138 (99.3%)	1.0 (ref)	1.0 (ref)
Yes	143 (1.7%)	1 (0.7%)	0.41 (0.06–2.94) p = 0.373	0.79 (0.10–6.43) p = 0.827
*Axillary temperature ≥ 37.5 °C or reported fever within previous 24 h*				
No	7971 (96.3%)	99 (71.2%)	1.0 (ref)	1.0 (ref)
Yes	305 (3.7%)	40 (28.8%)	12.00 (8.15–17.68) p < 0.001	13.64 (8.18–22.74) p < 0.001
*Used anti-malarial in previous month*				
No	8243 (99.6%)	135 (97.1%)	1.0 (ref)	1.0 (ref)
Yes	33 (0.4%)	4 (2.9%)	8.28 (2.87–23.89) p < 0.001	4.11 (1.06–15.90) p < 0.040
***Household-level risk factors***				
*> 1 RDT positives in household*				
No	8026 (97.0%)	77 (55.4%)	1.0 (ref)	1.0 (ref)
Yes	250 (3.0%)	62 (44.6%)	34.05 (23.65–49.01) p < 0.001	21.72 (13.83–34.10) p < 0.001
*Other members of household travelled in previous month*				
No	6712 (81.1%)	126 (90.6%)	1.0 (ref)	1.0 (ref)
Yes	1564 (18.9%)	13 (9.4%)	0.44 (0.25–0.78) p = 0.005	0.90 (0.46–1.74) p = 0.746
***Health facility catchment area***				
Dounde	946 (11.4%)	7 (5.0%)	1.0 (ref)	1.0 (ref)
Salalatou	1410 (17.0%)	86 (61.9%)	8.71 (4.01–18.91) p < 0.001	6.74 (2.36–19.23) p < 0.001
Oudalaye	929 (11.2%)	10 (7.2%)	1.46 (0.55–3.85) p = 0.445	2.25 (0.58–8.72) p = 0.242
Niaghana Tidel	666 (8.0%)	14 (10.1%)	2.88 (1.16–7.18) p = 0.023	1.64 (0.49–5.50) p = 0.421
Mbem mbem	1726 (20.9%)	11 (7.9%)	0.86 (0.33–2.23) p = 0.757	1.09 (0.32–3.73) p = 0.892
Gassane	2599 (31.4%)	11 (7.9%)	0.57 (0.22–1.48) p = 0.247	0.58 (0.17–1.93) p = 0.373
***Constant***				0.00 (0.00–0.01) p < 0.001
Total	8276	139	8276	8276

**Table 3:** Logistic regression results to assess the risk factors associated with RDT positivity: Individuals ≥ 10 years old

Covariates	N (% of total population ≥ 10 years)	n (distribution of RDT-positive population (%) ≥ 10 years)	Univariable odds ratio (95% CI) p-value	Multivariable adjusted odds ratio (95% CI) p-value
***Sociodemographic characteristics***				
*Sex*				
Female	6209 (58.2%)	59 (40.1%)	1.0 (ref)	1.0 (ref)
Male	4455 (41.8%)	88 (59.9%)	2.10 (1.51–2.93) p < 0.001	1.93 (1.10–3.36) p = 0.021
*Age (years)*				
10–14 years	2420 (22.7%)	67 (45.6%)	1.0 (ref)	1.0 (ref)
15–19 years	1731 (16.2%)	27 (18.4%)	0.56 (0.35–0.87) p = 0.011	0.67 (0.38–1.17) p = 0.156
20–29 years	2601 (24.4%)	22 (15.0%)	0.30 (0.18–0.49) p < 0.001	0.31 (0.17–0.56) p < 0.001
30–39 years	1588 (14.9%)	14 (9.5%)	0.31 (0.17–0.56) p < 0.001	0.36 (0.18–0.72) p = 0.004
40–49 years	923 (8.7%)	6 (4.1%)	0.23 (0.10–0.53) p < 0.001	0.22 (0.08–0.56) p = 0.002
50–59 years	644 (6.0%)	4 (2.7%)	0.22 (0.08–0.60) p = 0.003	0.20 (0.07–0.63) p = 0.006
60+ years	757 (7.1%)	7 (4.8%)	0.33 (0.15–0.72) p = 0.005	0.33 (0.14–0.79) p = 0.013
*Education*				
None/illiterate	8635 (81.0%)	119 (81.0%)	1.0 (ref)	1.0 (ref)
Primary/Arabic school	1784 (16.7%)	21 (14.3%)	0.85 (0.53–1.36) p = 0.503	0.65 (0.33–1.30) p = 0.227
Secondary/university	227 (2.1%)	6 (4.1%)	1.94 (0.85–4.46) p = 0.117	6.12 (1.97–19.08) p = 0.002
Other	18 (0.2%)	1 (0.7%)	4.21 (0.56–31.89) p = 0.164	7.05 (0.86–57.67) p = 0.068
*Occupation*				
Farmer/shepherd	3368 (31.6%)	52 (35.4%)	1.0 (ref)	1.0 (ref)
Student	982 (9.2%)	12 (8.2%)	0.79 (0.42–1.48) p = 0.462	0.70 (0.27–1.85) p = 0.473
Housekeeper	4315 (40.5%)	33 (22.4%)	0.49 (0.32–0.76) p < 0.001	0.84 (0.40–1.74) p = 0.633
Unemployed	1633 (15.3%)	43 (29.3%)	1.72 (1.15–2.59) p = 0.009	1.25 (0.70–2.25) p = 0.447
Other	366 (3.4%)	7 (4.8%)	1.24 (0.56–2.76) p = 0.592	1.18 (0.44–3.15) p = 0.738
***Individual malaria risk factors***				
*Used a bed net on previous night*				
No	3893 (36.5%)	55 (37.4%)	1.0 (ref)	1.0 (ref)
Yes	6771 (63.5%)	92 (62.6%)	0.96 (0.69–1.35) p = 0.818	0.77 (0.51–1.18) p = 0.233
*Travelled outside of district in previous two weeks*				
No	10,315 (96.7%)	144 (98.0%)	1.0 (ref)	1.0 (ref)
Yes	349 (3.3%)	3 (2.0%)	0.61 (0.19–1.93) p = 0.403	0.82 (0.18–3.81) p = 0.805
*Axillary temperature ≥ 37.5 °C or reported fever within previous 24 h*				
No	10,319 (96.8%)	101 (68.7%)	1.0 (ref)	1.0 (ref)
Yes	345 (3.2%)	46 (31.3%)	15.56 (10.78–22.47) p < 0.001	24.75 (15.20–40.30) p < 0.001
*Used anti-malarial in previous month*				
No	10,610 (99.5%)	138 (93.9%)	1.0 (ref)	1.0 (ref)
Yes	54 (0.5%)	9 (6.1%)	15.18 (7.28–31.65) p < 0.001	8.67 (3.30–22.77) p < 0.001
***Household-level risk factors***				
*> 1 RDT positives in household*				
No	10,349 (97.0%)	88 (59.9%)	1.0 (ref)	1.0 (ref)
Yes	315 (3.0%)	59 (40.1%)	26.87 (18.89–38.22) p < 0.001	20.10 (12.90–31.31) p < 0.001
*Other members of household travelled outside of district in previous two weeks*				
No	8430 (79.1%)	132 (89.8%)	1.0 (ref)	1.0 (ref)
Yes	2234 (20.9%)	15 (10.2%)	0.42 (0.25–0.73) p = 0.002	0.62 (0.32–1.20) p = 0.159
***Health facility catchment area***				
Dounde	1321 (12.4%)	13 (8.8%)	1.0 (ref)	1.0 (ref)
Salalatou	1703 (16.0%)	57 (38.8%)	3.48 (1.90–6.39) p < 0.001	2.53 (1.18–5.43) p = 0.017
Oudalaye	1012 (9.5%)	14 (9.5%)	1.41 (0.66–3.02) p = 0.374	2.16 (0.83–5.57) p = 0.113
Niaghana tidel	3602 (33.8%)	18 (12.2%)	3.03 (1.55–5.93) p < 0.001	2.02 (0.86–4.75) p = 0.107
Mbem mbem	889 (8.3%)	26 (17.7%)	0.90 (0.44–1.83) p = 0.777	1.03 (0.43–2.47) p = 0.940
Gassane	2137 (20.0%)	19 (12.9%)	0.51 (0.25–1.03) p = 0.062	0.43 (0.18–1.03) p = 0.058
***Constant***				0.01 (0.00–0.02) p < 0.001
Total	10,664	147	10,664	10,664

RDT positivity showed evidence of clustering at the household level, with 43% of the total infections being in households with at least one other infection and 90% of households not having any RDT positives.

### Impact evaluation

**Fig. 4:**
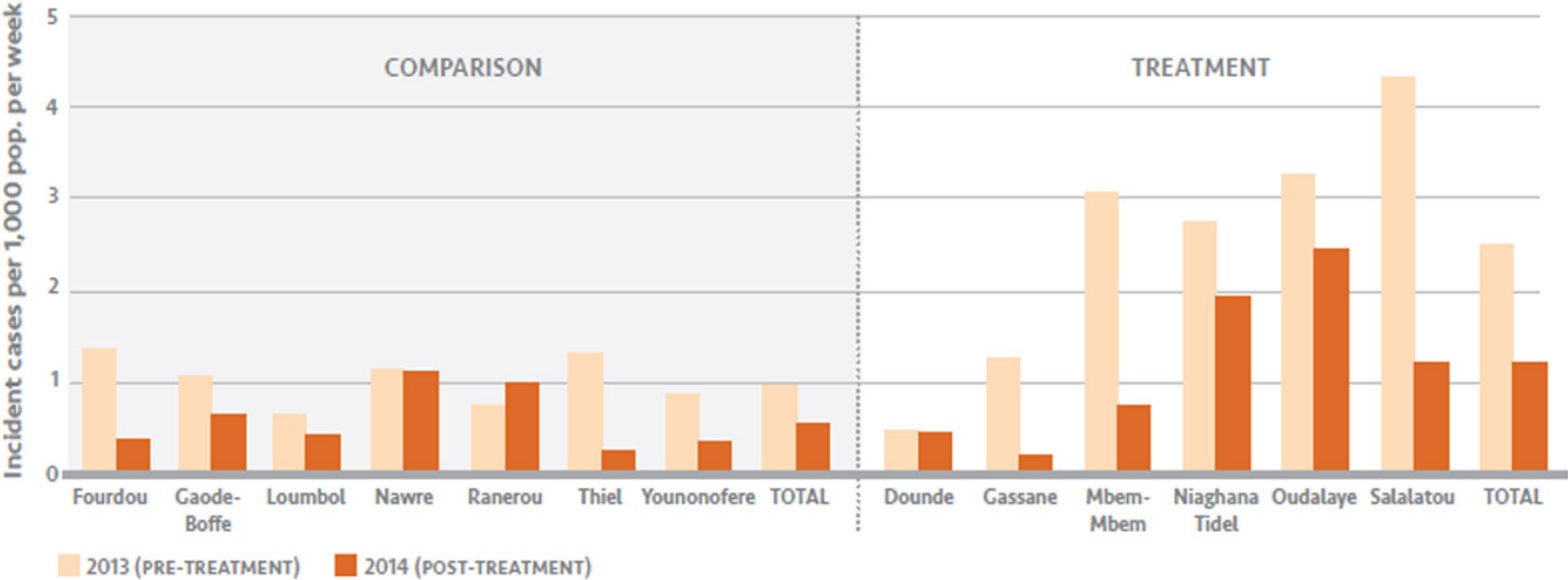
Average incident malaria cases per week pre- and post-intervention by HFCA

Figure 4 shows the average incident malaria cases per week in the intervention and comparison groups before and after the intervention, and Table 4 shows the results of the difference-in-differences analysis. The pre/post variable was not significant, showing that malaria rates did not change significantly in the comparison group, and the comparison/intervention variables were also not significant, showing that on average malaria cases were not significantly different between the comparison and intervention groups at baseline when controlling for other factors. However, the interaction between the pre/post and comparison/intervention variables had an adjusted incidence risk ratio (IRR) of 0.62 (95% confidence interval 0.45–0.84, p = 0.002), so the IRR of post versus pre-intervention period was 0.87 in the comparison group and 0.54 in the intervention group. Thus there was a significant 38% larger decrease in the average number of malaria cases between the pre and the post intervention periods in the intervention group relative to that in the comparison group, after adjusting for rainfall, NDVI, average bed net ownership and household size.

**Table 4:** Differences-in-differences analysis using negative binomial regression on malaria cases

Covariates	Incidence risk ratio (95% CI)	*p* value
Time effects		
Pre-intervention	1.0 (Ref.)	–
Post-intervention	0.87 (0.69–1.09)	0.232
Group effects		
Comparison group	1.0 (Ref.)	–
Treated group	0.93 (0.65–1.34)	0.697
Intervention effect (interaction term)	0.62** (0.45–0.84)	0.002
HFCA-level variables		
Average number of bed nets per sleeping space	4.96* (1.45–16.99)	0.011
Average number of people per household	0.95* (0.90–1.00)	0.038
Environmental variables		
Rainfall (2 mo. lag)	1.01** (1.01–1.02)	<0.001
NDVI (2 mo. lag)	1.05** (1.04–1.06)	<0.001
Constant	0.00** (0.00–0.00)	<0.001
Observations	542	
Number of HFCAs	13	

* p < 0.05, ** p < 0.01

In a sensitivity analysis, the significance of the effect decreased when including the RDT-positive febrile individuals found during the MTAT and PECADOM in the outcome (IRR = 0.76, 95% CI 0.56–1.04, p = 0.083). Since the coefficient on average bed net ownership was the opposite as expected, an additional sensitivity analysis was run omitting this variable from the regression, and found that the interaction term remained similar to the original analysis (IRR = 0.64, 95% CI 0.47–0.87, p = 0.004).

Additional file 2a and b show the weekly incidence of malaria cases per 1000 population during the high malaria transmission season in intervention and comparison HFCAs before and after the intervention. Additional file 5 shows the traditional difference-in-differences diagram including the 2012–2013 malaria season incidence data (except the HFCA of Doundé, for which data from that initial season were missing), to assess the parallel trends assumption. The incidence trends during the pre-intervention period in the intervention and comparison groups suggest the parallel trends assumption holds true.

### Costing

The cost for MTAT was $14.3 per person tested. The bulk of the cost was incurred during implementation, which was 85% of the total and was largely made up of supervision costs (38% of total) and transportation costs (19% of total). The cost for PECADOM++ was lower, at $3.1 per person. Supervisor and transportation costs were a lower share of costs than in the MTAT and made up 18% and 22% of total costs, respectively, while training costs made up the largest share at 33%. Case investigation was by far the cheapest intervention at $0.4 per person. Training (29%), transportation (28%), and supervision (25%) were again the dominant cost categories. The salaries of enumerators and community health workers was a small fraction of the costs, less than 20% for all interventions. Costing details for MTAT, PECADOM++ and case investigation are shown in Additional files 6, 7 and 8, respectively.

## Discussion

The study demonstrated the feasibility of implementing a large-scale MTAT in 4 weeks, followed by PECADOM++ and case investigation during the rest of the transmission season in a low to moderate malaria transmission setting. The MTAT achieved a household coverage of 89% and a within household coverage of 87% for a combined effective coverage of 77% for RDT testing, which is lower than the 88% reported in the Zambia MTAT [7], but higher than the 64% reported in Zanzibar [8]. Males 15–49 years old were found to be a disproportionately large part of the absent household members, likely due to absence for employment. This indicates that alternative delivery strategies, like visiting work sites or visiting homes outside of working hours, would be needed to be able to reach these men. Treatment with DHAp was safe and adherence was high. PECADOM++ was also implemented with a good coverage, although it varied largely by HFCA and week. In half of the HFCAs it took a few weeks to achieve high coverage of households. Nevertheless, stratifying the intervention at the village level created difficulties both in defining exact geographic boundaries and in supervising the different interventions within each HFCA. If, instead, stratification of the interventions had been done at the HFCA-level it would have made it easier to supervise and determine each household’s intervention assignment.

In the study area, the MTAT approach resulted in a very small proportion of the population receiving antimalarial treatment, as only 291 (1.5%) of the 18,992 individuals tested were positive. However, in an area with such low malaria transmission intensity, a substantial number of infections may have been low density and thus not detectable by RDTs [18–20]. The usefulness and cost effectiveness of highly sensitive RDTs could be assessed for MTAT.

During PECADOM++, 40,002 visits were needed to find 2784 individuals with reported fever and treat a total of 289 individuals. This screening programme was time and labour intensive considering the number of cases found. In southern Senegal, this approach yielded a much higher RDT positivity rate, with 62% of the tested febrile individuals having a positive RDT [21]. Thus, pursuing the PECADOM++ strategy in the low transmission areas of the north may be difficult to justify. RDT positivity was higher than during the MTAT, as during PECADOM++ only individuals with reported fever in the previous week were tested. Conversely though, the MTAT revealed many afebrile infections (of the 291 positive cases found during MTAT, only 106 (36.4%) had a measured fever or reported fever in the previous week), suggesting that the PECADOM++ approach, which relies on fever screening, likely missed many afebrile infections. There was limited power to detect differences between age groups but the lack of significant differences by age group challenges the notion that natural immunity takes a long time to develop and most infections, especially in young children, cause fever in low transmission settings [22]. This is in agreement with some other studies in low transmission settings [18], suggesting that interventions that only target febrile individuals will not clear all infections. Of note, this assessment was cross sectional in nature, and thus had limited ability to detect the appearance of fever or other symptoms over time. In the MTAT, the age groups that had the highest RDT positivity were participants aged 5–19 years. Moreover, in the risk factor analysis those aged 5–9 and 10–14 years old had higher odds of being positive, suggesting that older children and young adults may have more exposure to mosquitoes. Overall, the interaction between transmission intensity, age and immunity is still poorly understood in very low transmission settings, especially in areas where transmission dropped and there is still naturally acquired immunity in the community, and more evidence is needed to understand the development of naturally acquired immunity and if screening for a more comprehensive panel of symptoms would capture all or most infections.

The risk factor analysis also showed that, as expected, fever and having taken an anti-malarial in the previous month were risk factors for RDT positivity in both age groups, whereas bed net use the previous night was unexpectedly not significant. Having at least one other RDT-positive in the household was also a risk factor in both age groups, which, together with the fact that 43% of the total infections were in households with at least one other infection and 90% of households did not have any RDT positives, indicates clustering of infections at the household level. On the other hand, during the PECADOM++, only 17.3% of infections occurred in households that had other RDT positives during the study period, showing that infections do not always occur in the same households over a transmission season and that strategies that try to target hotspots only will miss a large proportion of the infections. In children ≥ 10 years old and adults, having had primary education or Arabic school was found to be protective relative to no education, whereas secondary education was a risk factor, which could be due to higher educated individuals travelling more to higher transmission areas or other behavioral differences.

The combination of interventions resulted in an estimated 38% (95% CI 16–55%) larger decrease in malaria case incidence in the intervention group relative to that in the comparison group. This is a significant reduction and suggests that MTAT in combination with PECADOM++ and case investigation offer a feasible parasite reduction strategy. However, this reduction in incidence was moderate and it was achieved with intensive effort. Both MTAT and PECADOM++ required supervision, transportation, training field teams and conducting large-scale individual testing at significant expense. Nevertheless, this impact is higher than that found in previous MTAT studies, where no follow up interventions like PECADOM++ or case investigation were done after the MTAT. A study conducted in Zambia found a 53% decrease on the prevalence of infection with a marginal effect on outpatient malaria case incidence [7]. Two other studies in Zanzibar and Burkina Faso showed no effect on the incidence of malaria [8], 9]. Both of these studies noted a high prevalence of low density infections not detected by RDT, which may be a major driver of the ineffectiveness of MTAT programmes. Based on evidence from these previous studies, the World Health Organization does not currently recommend the use of MTAT to interrupt malaria transmission [23].

The costs for these interventions must also be considered. The cost of $13.9 per person reached is notably higher than the cost of other malaria control interventions [24]. However, it is likely that costs could be substantially reduced in programmatic implementation, particularly with the reduction of supervision and transportation costs. Also, the study area covered a large geographic area with low density of villages and population, requiring significantly more travel time than would be needed in higher density settings. The salaries of enumerators and community health workers was a small fraction of the costs, less than 20% for all interventions, suggesting that if extraneous costs could be reduced and training routinized, costs could be significantly reduced. A formal cost-effectiveness study would be necessary to determine how this intervention could fit in with others.

The quasi-experimental design and impact evaluation analysis have a few caveats. First, while the difference-in-differences framework allows for comparison between groups with different baseline levels and analysis suggests that the parallel trends assumption holds true in this case, the intervention group was selected based on the higher intensity of malaria transmission in prior years, a programmatic decision which complicated the estimation of impact. Second, this study featured a small number of clusters with selection done at the HFCA level. Third, while reductions in rainfall and NDVI during the intervention period were associated with declines in malaria incidence in both groups, these changes in rainfall and NDVI might have had a different effect on malaria transmission in the intervention than in the comparison areas, given the baseline differences. Fourth, the study used passively collected data on malaria case incidence, assuming that the interventions did not affect care-seeking behaviour and tested this assumption in a sensitivity analysis. When adding the febrile RDT positives found during the MTAT and PECADOM++ in the outcome, the effect of the intervention was smaller and became non-significant. This sensitivity analysis assumed that all febrile individuals would have sought care, however, other studies in the north of Senegal show that only 60% of febrile children younger than 5 years seek care from a health facility or community health worker [25]. Therefore, the number of children that would have sought care in the absence of the study was likely smaller and the IRR of this sensitivity analysis is underestimated. Finally, the regression showed that household ownership of a mosquito net was associated with a higher incidence of malaria, although this likely reflects higher number of nets distributed or higher usage of ITNs in areas with higher transmission and an additional sensitivity analysis showed that this variable had little impact on the main outcome.

## Conclusion

It is operationally feasible to conduct MTAT and PECADOM++ with a high coverage. PECADOM++ was not an efficient strategy to complement the MTAT, as maintaining a high coverage every week required substantial resources to find and treat a small number of infections. MTAT, implemented at the beginning of the transmission season and followed with PECADOM++ and case investigation, showed only a modest impact. This study suggests alternative or additional strategies are needed to eliminate the parasite reservoir.

## Supplementary information

**Additional file 1.** Distribution of independent and dependent variables.

**Additional file 2.** Weekly incident malaria cases per 1000 population during the high transmission seasons (Sept. to January) in a) intervention and b) comparison health facility catchment areas*.

**Additional file 3.** PECADOM++ profile.

**Additional file 4.** Weekly PECADOM++ household coverage by health facility catchment area.

**Additional file 5.** Incident malaria cases over high malaria transmission seasons (Sept. to January) of 2012–2013*, 2013–2014 and 2014–2015 in intervention vs comparison group to assess parallel trends assumption in the difference-in-difference analysis.

**Additional file 6.** MTAT costs

**Additional file 7.** PECADOM++ costs.

**Additional file 8.** Case investigation costs.

## Data Availability

Data can be requested from the investigators.

## Abbreviations

AL: Artemether-lumefantrine
DHAp: Dihydro-artemisinin-piperaquine
FTAT: Focal test and treat
FSTAT: Focal screen, test and treat
HFCA: Health facility catchment area
LLIN: Long-lasting insecticide-treated net
MACEPA: Malaria control and elimination partnership in Africa
MDA: Mass drug administration
MTAT: Mass test and treat
NDVI: Normalized difference vegetation index
PECADOM: *Prise en charge à domicile* (case management at the household level)
PNLP: *Programme National de Lutte contre le Paludisme* (National malaria control programme)
RDT: Rapid diagnostic test

## References

[1] Feachem RG, Phillips AA, Hwang J, Cotter C, Wielgosz B, Greenwood BM (2010). Shrinking the malaria map: progress and prospects. Lancet.

[2] Newby G, Bennett A, Larson E, Cotter C, Shretta R, Phillips AA (2016). The path to eradication: a progress report on the malaria-eliminating countries. Lancet.

[3] Thwing JI, Perry RT, Townes DA, Diouf MB, Ndiaye S, Thior M (2011). Success of Senegal’s first nationwide distribution of long-lasting insecticide-treated nets to children under five—contribution toward universal coverage. Malar J..

[4] The DHS Program—Senegal: Malaria Indicator Survey (MIS), 2008. http://dhsprogram.com/what-we-do/survey/survey-display-338.cfm. Accessed Jan 13 2017.

[5] Agence Nationale de la Statistique et de la Démographie, the DHS program ICF. Sénégal: Enquête Démographique et de Santé Continue 2017. 2018. https://dhsprogram.com/pubs/pdf/FR345/FR345.pdf.

[6] Okell LC, Griffin JT, Kleinschmidt I, Hollingsworth TD, Churcher TS, White MJ (2011). The potential contribution of mass treatment to the control of *Plasmodium falciparum* malaria. PLoS ONE.

[7] Larsen DA, Bennett A, Silumbe K, Hamainza B, Yukich JO, Keating J (2015). Population-wide malaria testing and treatment with rapid diagnostic tests and artemether-lumefantrine in southern Zambia: a community randomized step-wedge control trial design. Am J Trop Med Hyg.

[8] Cook J, Xu W, Msellem M, Vonk M, Bergström B, Gosling R (2015). Mass screening and treatment on the basis of results of a *Plasmodium falciparum*-specific rapid diagnostic test did not reduce malaria incidence in Zanzibar. J Infect Dis.

[9] Tiono AB, Ouédraogo A, Ogutu B, Diarra A, Coulibaly S, Gansané A (2013). A controlled, parallel, cluster-randomized trial of community-wide screening and treatment of asymptomatic carriers of *Plasmodium falciparum* in Burkina Faso. Malar J..

[10] Linard C, Gilbert M, Snow RW, Noor AM, Tatem AJ (2012). Population distribution, settlement patterns and accessibility across Africa in 2010. PLoS ONE.

[11] Trape JF, Rogier C, Konate L, Diagne N, Bouganali H, Canque B (1994). The Dielmo project: a longitudinal study of natural malaria infection and the mechanisms of protective immunity in a community living in a holoendemic area of Senegal. Am J Trop Med Hyg.

[12] Sinka ME, Bangs MJ, Manguin S, Rubio-Palis Y, Chareonviriyaphap T, Coetzee M (2012). A global map of dominant malaria vectors. Parasit Vectors..

[13] Agence Nationale de la Statistique et de la Démographie ICF. Sénégal Enquête Démographique et de Santé Continue (EDS-Continue) 2014. 2015. https://www.dhsprogram.com/publications/publication-FR305-DHS-Final-Reports.cfm. Accessed May 3 2019.

[14] Programme National de Lutte contre le Paludisme (2013). Directives nationales de prévention et de prise en charge du paludisme.

[15] Khandker SR, Koolwal GB, Samad HA (2009). Handbook on impact evaluation: quantitative methods and practices.

[16] Early Warning and Environmental Monitoring Program. https://earlywarning.usgs.gov/fews/search/Africa/West%20Africa. Accessed Jan 13 2017.

[17] Larson BA, Ngoma T, Silumbe K, Rutagwera M-RI, Hamainza B, Winters AM (2016). A framework for evaluating the costs of malaria elimination interventions: an application to reactive case detection in Southern Province of Zambia, 2014. Malar J.

[18] Okell LC, Bousema T, Griffin JT, Ouédraogo AL, Ghani AC, Drakeley CJ (2012). Factors determining the occurrence of submicroscopic malaria infections and their relevance for control. Nat Commun..

[19] Schachterle SE, Mtove G, Levens JP, Clemens EG, Shi L, Raj A (2011). Prevalence and density-related concordance of three diagnostic tests for malaria in a region of Tanzania with hypoendemic malaria. J Clin Microbiol.

[20] Ranadive N, Kunene S, Darteh S, Ntshalintshali N, Nhlabathi N, Dlamini N (2017). Limitations of rapid diagnostic testing in patients with suspected malaria: a diagnostic accuracy evaluation from Swaziland, a low-endemicity country aiming for malaria elimination. Clin Infect Dis.

[21] Linn AM, Ndiaye Y, Hennessee I, Gaye S, Linn P, Nordstrom K (2015). Reduction in symptomatic malaria prevalence through proactive community treatment in rural Senegal. Trop Med Int Health..

[22] Doolan DL, Dobano C, Baird JK (2009). Acquired immunity to malaria. Clin Microbiol Rev.

[23] Global Malaria Programme (2015). The role of mass drug administration, mass screening and treatment, and focal screening and treatment for malaria.

[24] White MT, Conteh L, Cibulskis R, Ghani AC (2011). Costs and cost-effectiveness of malaria control interventions–a systematic review. Malar J..

[25] Agence Nationale de la Statistique et de la Démographie, Sénégal. Sénégal: Enquête Démographique et de Santé Continue (EDS-Continue) 2016. The DHS Program, Maryland, USA; 2017.

