# Correction to: Mass testing and treatment for malaria followed by weekly fever screening, testing and treatment in Northern Senegal: feasibility, cost and impact

**DOI:** 10.1186/s12936-020-03484-2

**Published:** 2020-11-30

**Authors:** Ruben O. Conner, Yakou Dieye, Michael Hainsworth, Adama Tall, Badara Cissé, Farba Faye, Mame Demba Sy, Amadou Ba, Doudou Sene, Souleymane Ba, Elhadji Doucouré, Tidiane Thiam, Moussa Diop, Kammerle Schneider, Moustapha Cissé, Mady Ba, Callie A. Scott, Ritu Kumar, Elias Asfaw, Duncan Earle, Philippe Guinot, Richard W. Steketee, Caterina Guinovart

**Affiliations:** 1 PATH Malaria Control and Elimination Partnership in Africa (MACEPA), 2201 Westlake Avenue, Suite 200, Seattle, WA 98121 USA; 2 Programme National de Lutte contre le Paludisme (PNLP), Ministère de la Santé et l’Action Sociale, Dakar, Senegal; 3 Barcelona Institute for Global Health (ISGlobal), Hospital Clínic–Universitat de Barcelona, Rosselló 132, 08036 Barcelona, Spain

## Correction to: Malar J 19:252 (2020) 10.1186/s12936-020-03313-6

Following publication of the original article [1], the authors reported an error in the author list of the article.

Namely, the following three authors have been omitted from the list in error: Callie A. Scott, Ritu Kumar and Elias Asfaw.

These authors worked on the costing that is presented in the article’s supplementary material and so should be included in the author list.

Please find the corrected list in this correction.

The authors apologize for any inconvenience caused.
