# Supplementary material for: Mass testing and treatment for malaria followed by weekly fever screening, testing and treatment in Northern Senegal: feasibility, cost and impact
Source: Malar J. 2020 Jul 14;19:252. doi: 10.1186/s12936-020-03313-6 (PMC7362450; doi:10.1186/s12936-020-03313-6)
Supplement: Supplementary file 1 — Additional file 1. Distribution of independent and dependent variables. [file 12936_2020_3313_MOESM1_ESM.docx]

# **Additional figures and tables**

Additional File 1. Distribution of independent and dependent variables

|  | **Variable** | **Obs** | **Pre mean** | **Post mean** | **Pre std. dev.** | **Post std. dev.** | **Pre min** | **Post min** | **Pre max** | **Post max** |
| --- | --- | --- | --- | --- | --- | --- | --- | --- | --- | --- |
| Comparison group | Malaria cases | 146 | 6.82 | 3.27 | 9.55 | 3.83 | 0 | 0 | 55 | 17 |
|  | Bed nets per sleeping space* (Avg #) | 146 | 0.26 | 0.26 | 0.08 | 0.08 | 0 | 0 | 0 | 0 |
|  | People per household* (Avg #) | 146 | 9.13 | 9.13 | 2.92 | 2.92 | 7 | 7 | 16 | 16 |
|  | Rainfall (2 month lag) | 146 | 27.36 | 22.59 | 19.79 | 18.80 | 0 | 0 | 109 | 85 |
|  | NDVI (2 month lag) | 146 | 133.65 | 130.34 | 10.11 | 8.21 | 118 | 117 | 157 | 153 |
| Intervention group | Malaria cases | 125 | 13.91 | 6.06 | 17.00 | 8.71 | 0 | 0 | 81 | 47 |
|  | Bed nets per sleeping space* (Avg #) | 125 | 0.42 | 0.42 | 0.23 | 0.23 | 0 | 0 | 1 | 1 |
|  | People per household* (Avg #) | 125 | 11.98 | 11.98 | 3.47 | 3.47 | 8 | 8 | 18 | 18 |
|  | Rainfall (2 month lag) | 125 | 30.08 | 26.29 | 23.04 | 20.25 | 0 | 0 | 141 | 98 |
|  | NDVI (2 month lag) | 125 | 141.23 | 137.46 | 11.54 | 10.36 | 121 | 120 | 164 | 161 |

*these variables were only measured at baseline and were assumed not to change over the course of the study
