# Supplementary material for: Mass testing and treatment for malaria followed by weekly fever screening, testing and treatment in Northern Senegal: feasibility, cost and impact
Source: Malar J. 2020 Jul 14;19:252. doi: 10.1186/s12936-020-03313-6 (PMC7362450; doi:10.1186/s12936-020-03313-6)
Supplement: Supplementary file 2 — Additional file 2. Weekly incident malaria cases per 1000 population during the high transmission seasons (Sept. to January) in a) intervention and b) comparison health facility catchment areas*. [file 12936_2020_3313_MOESM2_ESM.docx]

Additional File 2a & 2b. Weekly incident malaria cases per 1000 population during the high transmission seasons (Sept. to January) in a) intervention and b) comparison health facility catchment areas*

*Incidence data was unavailable for Doundé in the 2012-2013 period
