# Supplementary material for: Mass testing and treatment for malaria followed by weekly fever screening, testing and treatment in Northern Senegal: feasibility, cost and impact
Source: Malar J. 2020 Jul 14;19:252. doi: 10.1186/s12936-020-03313-6 (PMC7362450; doi:10.1186/s12936-020-03313-6)
Supplement: Supplementary file 3 — Additional file 3. PECADOM++ profile. [file 12936_2020_3313_MOESM3_ESM.docx]

Additional File 3. PECADOM++ profile

Total HHs in targeted villages: 3,577

93% (3307) of HHs approached for PECADOM++ at least once with 40,002 visits during 16 weeks

9.4% of individuals tested during the FTAT had a positive RDT (119/1,271)

1,271 individuals tested during the FTAT in RDT-positive HHs

6.5% of fever cases had a positive RDT (170/2,612)

36,481 visits could be completed, of which 5.2% (1,889) found at least one fever case: 2784 individuals with fever
