# Supplementary figures and images for: Mass testing and treatment for malaria followed by weekly fever screening, testing and treatment in Northern Senegal: feasibility, cost and impact
Source: Malar J. 2020 Jul 14;19:252. doi: 10.1186/s12936-020-03313-6 (PMC7362450; doi:10.1186/s12936-020-03313-6)

Additional File 4. Weekly PECADOM++ household coverage by health facility catchment area

**
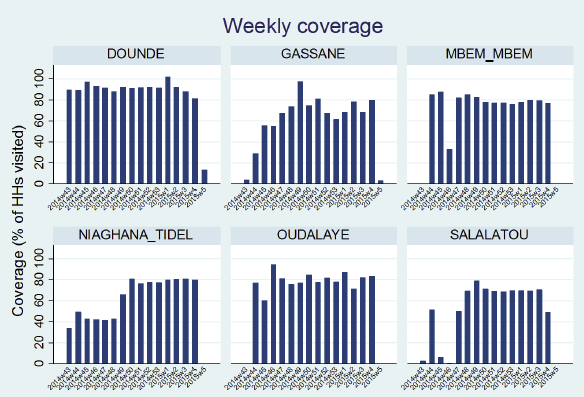
**

Supplement: Supplementary file 4 — Additional file 4. Weekly PECADOM++ household coverage by health facility catchment area. [file 12936_2020_3313_MOESM4_ESM.docx]
