# Supplementary material for: Mass testing and treatment for malaria followed by weekly fever screening, testing and treatment in Northern Senegal: feasibility, cost and impact
Source: Malar J. 2020 Jul 14;19:252. doi: 10.1186/s12936-020-03313-6 (PMC7362450; doi:10.1186/s12936-020-03313-6)
Supplement: Supplementary file 5 — Additional file 5. Incident malaria cases over high malaria transmission seasons (Sept. to January) of 2012–2013*, 2013–2014 and 2014–2015 in intervention vs comparison group to assess parallel trends assumption in the difference-in-difference analysis. [file 12936_2020_3313_MOESM5_ESM.docx]

Additional File 5. Incident malaria cases over high malaria transmission seasons (Sept. to January) of 2012-13*, 2013-14 and 2014-15 in intervention vs comparison group to assess parallel trends assumption in the difference-in-difference analysis

*There are missing data from one intervention HFCA (Doundé) for the 2012-2013 season
