# Supplementary material for: Mass testing and treatment for malaria followed by weekly fever screening, testing and treatment in Northern Senegal: feasibility, cost and impact
Source: Malar J. 2020 Jul 14;19:252. doi: 10.1186/s12936-020-03313-6 (PMC7362450; doi:10.1186/s12936-020-03313-6)
Supplement: Supplementary file 6 — Additional file 6. MTAT costs [file 12936_2020_3313_MOESM6_ESM.docx]

Additional File 6. MTAT Costs

| **Outputs** | | |  |  |  | |  |  |  |  |
| --- | --- | --- | --- | --- | --- | --- | --- | --- | --- | --- |
|  |  |  |  |  |  | |  |  |  |  |
|  | Output | | Total | Total per 1000 population |  | |  |  |  |  |
|  | Population | | 21,911 | 22 |  | |  |  |  |  |
|  | Number of villages covered | | 46 | 2.10 |  | |  |  |  |  |
|  | Number of households visited | | 2,225 | 102 |  | |  |  |  |  |
|  | Number of individuals tested | | 18,992 | 867 |  | |  |  |  |  |
|  | Number of individuals treated | | 263 | 12 |  | |  |  |  |  |
|  |  |  |  |  |  | |  |  |  |  |
| **Total cost per year** | | |  |  |  | |  |  |  |  |
|  |  |  |  |  |  | |  |  |  |  |
|  | Cost category | | Total cost | | Share of total costs |  |  |  |  |  |
|  |  |  | 2014 XOF | 2014 USD |  |  |  |  |  |  |
|  | Total preparation costs | | 9,161,718 | 18,534 | 6.8% |  |  |  |  |  |
|  | Total training costs | | 10,826,370 | 21,901 | 8.0% |  |  |  |  |  |
|  | Total implementation costs | | 114,703,423 | 232,042 | 85.2% |  |  |  |  |  |
|  |  | *CHW and enumerator pairs, salaries and DSA* | *15,332,727* | *31,018* | *11.4%* |  |  |  |  |  |
|  |  | *Health facility staff, salaries and DSA* | *10,637,932* | *21,520* | *7.9%* |  |  |  |  |  |
|  |  | *Supervisors, salaries and DSA* | *51,193,797* | *103,564* | *38.0%* |  |  |  |  |  |
|  |  | *Transportation* | *25,974,689* | *52,546* | *19.3%* |  |  |  |  |  |
|  |  | *Mobile phones and accessories* | *307,605* | *622* | *0.2%* |  |  |  |  |  |
|  |  | *RDTs* | *3,379,732* | *6,837* | *2.5%* |  |  |  |  |  |
|  |  | *Malaria treatment (DHAP and AL)* | *86,432* | *175* | *0.1%* |  |  |  |  |  |
|  |  | *Other supplies* | *7,790,508* | *15,760* | *5.8%* |  |  |  |  |  |
|  | Total costs | | 134,691,511 | 272,478 | 100.0% |  |  |  |  |  |
|  |  |  |  |  |  | |  |  |  |  |
| **Cost per output** | | |  |  |  | |  |  |  |  |
|  |  |  |  |  |  | |  |  |  |  |
|  | Metric |  | 2014 XOF | 2014 USD |  | |  |  |  |  |
|  | Cost per household visited | | 60,536 | 122 |  | |  |  |  |  |
|  | Cost per individual tested | | 7,092 | 14.3 |  | |  |  |  |  |
|  | Cost per individual treated | | 512,135 | 1,036 |  | |  |  |  |  |
|  |  |  |  |  |  | |  |  |  |  |
