# Supplementary material for: Mass testing and treatment for malaria followed by weekly fever screening, testing and treatment in Northern Senegal: feasibility, cost and impact
Source: Malar J. 2020 Jul 14;19:252. doi: 10.1186/s12936-020-03313-6 (PMC7362450; doi:10.1186/s12936-020-03313-6)
Supplement: Supplementary file 7 — Additional file 7. PECADOM++ costs. [file 12936_2020_3313_MOESM7_ESM.docx]

Additional File 7. PECADOM++ Costs

| **Outputs** | | |  |  |  | | |  | |  | |  |  |
| --- | --- | --- | --- | --- | --- | --- | --- | --- | --- | --- | --- | --- | --- |
|  |  |  |  |  |  | | |  | |  | |  |  |
|  | **Output** | | **Total** | **Total per 1000 population** |  | | |  | |  | |  |  |
|  | Population | | 34,701 | 35 |  | | |  | |  | |  |  |
|  | Number of villages | | 52 | 1.50 |  | | |  | |  | |  |  |
|  | Number of household visits completed | | 36,481 | 1,051 |  | | |  | |  | |  |  |
|  | Number of individuals tested | | 2,612 | 75 |  | | |  | |  | |  |  |
|  | Number of individuals treated | | 161 | 2 |  | | |  | |  | |  |  |
|  |  |  |  |  |  | | |  | |  | |  |  |
|  |  |  |  |  |  | | |  | |  | |  |  |
| **Total cost per year** | | |  |  |  | | |  | |  | |  |  |
|  |  |  |  |  |  | | |  | |  | |  |  |
|  | Cost category | | Total cost | | Share of total costs |  |  |  |  |  |  |  |  |
|  |  |  | 2014 XOF | 2014 USD |  |  |  |  |  |  |  |  |  |
|  | Total preparation costs | | 2,290,430 | 4,633 | 4.1% |  |  |  |  |  |  |  |  |
|  | Total training costs | | 18,569,377 | 37,565 | 33.0% |  |  |  |  |  |  |  |  |
|  | Total implementation costs | | 35,475,143 | 71,765 | 63.0% |  |  |  |  |  |  |  |  |
|  |  | *CHW and enumerator pairs, salaries and DSA* | *9,597,273* | 19,415 | 17.0% |  |  |  |  |  |  |  |  |
|  |  | *Health facility staff, salaries and DSA* | *849,083* | 1,718 | 1.5% |  |  |  |  |  |  |  |  |
|  |  | *Supervisors, salaries and DSA* | *10,209,909* | 20,654 | 18.1% |  |  |  |  |  |  |  |  |
|  |  | *Transportation* | *12,420,182* | 25,126 | 22.0% |  |  |  |  |  |  |  |  |
|  |  | *Mobile phones and other equipment* | *53,434* | 108 | 0.1% |  |  |  |  |  |  |  |  |
|  |  | *RDTs* | *464,820* | 940 | 0.8% |  |  |  |  |  |  |  |  |
|  |  | *Malaria treatment (DHAP)* | *52,527* | 106 | 0.1% |  |  |  |  |  |  |  |  |
|  |  | *Other supplies* | *1,827,915* | 3,698 | 3.2% |  |  |  |  |  |  |  |  |
|  | Total costs | | 56,334,950 | 113,964 | 100.0% |  |  |  |  |  |  |  |  |
|  |  |  |  |  |  | |  | |  | |  |  |  |
|  |  |  |  |  |  | | |  | |  | |  |  |
| **Cost per output** | | |  |  |  | | |  | |  | |  |  |
|  |  |  |  |  |  | | |  | |  | |  |  |
|  | **Metric** | | **2014 XOF** | **2014 USD** |  | | |  | |  | |  |  |
|  | Cost per household visited | | 1,544 | 3.1 |  | | |  | |  | |  |  |
|  | Cost per individual tested | | 21,568 | 44 |  | | |  | |  | |  |  |
|  | Cost per individual treated | | 349,907 | 708 |  | | |  | |  | |  |  |
|  |  |  |  |  |  | | |  | |  | |  |  |
