# Supplementary material for: Mass testing and treatment for malaria followed by weekly fever screening, testing and treatment in Northern Senegal: feasibility, cost and impact
Source: Malar J. 2020 Jul 14;19:252. doi: 10.1186/s12936-020-03313-6 (PMC7362450; doi:10.1186/s12936-020-03313-6)
Supplement: Supplementary file 8 — Additional file 8. Case investigation costs. [file 12936_2020_3313_MOESM8_ESM.docx]

Additional File 8. Case Investigation Costs

| **Outputs** | | |  |  |  | |  | |  |  |  |  |
| --- | --- | --- | --- | --- | --- | --- | --- | --- | --- | --- | --- | --- |
|  |  |  |  |  |  | |  | |  |  |  |  |
|  | **Output** | | **Total** | **Total per 1000 population** |  | |  | |  |  |  |  |
|  | Population | | 192,616 | 193 |  | |  | |  |  |  |  |
|  | Number of health posts | | 31 | 0.16 |  | |  | |  |  |  |  |
|  | Number of households visited | | 1,180 | 6.13 |  | |  | |  |  |  |  |
|  | Number of individuals tested | | 8,424 | 43.74 |  | |  | |  |  |  |  |
|  | Number of individuals treated | | 229 | 1.19 |  | |  | |  |  |  |  |
|  |  |  |  |  |  | |  | |  |  |  |  |
| **Total cost per year** | | |  |  |  | |  | |  |  |  |  |
|  |  |  |  |  |  | |  | |  |  |  |  |
|  | Cost category | | Total cost | | Share of total costs |  | |  |  |  |  |  |
|  |  |  | 2014 XOF | 2014 USD |  |  | |  |  |  |  |  |
|  | Total preparation costs | | 2,290,430 | 4,633 | 3.0% |  | |  |  |  |  |  |
|  | Total training costs | | 22,174,055 | 44,858 | 29.1% |  | |  |  |  |  |  |
|  | Total implementation costs | | 51,773,349 | 104,736 | 67.9% |  | |  |  |  |  |  |
|  |  | *CHW and enumerator pairs, salaries and DSA* | *5,752,727* | *11,638* | *7.5%* |  | |  |  |  |  |  |
|  |  | *Health facility staff, salaries and DSA* | *3,847,918* | *7,784* | *5.0%* |  | |  |  |  |  |  |
|  |  | *Supervisors, salaries and DSA* | *18,662,732* | *37,754* | *24.5%* |  | |  |  |  |  |  |
|  |  | *Transportation* | *21,302,753* | *43,095* | *27.9%* |  | |  |  |  |  |  |
|  |  | *Mobile phone and accessories* | *134,116* | *271* | *0.2%* |  | |  |  |  |  |  |
|  |  | *RDTs* | *189,725* | *384* | *0.2%* |  | |  |  |  |  |  |
|  |  | *Malaria treatment (DHAP and AL)* | *12,488* | *25* | *0.0%* |  | |  |  |  |  |  |
|  |  | *Other supplies* | *1,870,890* | *3,785* | *2.5%* |  | |  |  |  |  |  |
|  | Total costs | | 76,237,833 | 154,227 | 100.0% |  | |  |  |  |  |  |
|  |  |  |  |  |  | |  | |  |  |  |  |
|  |  |  |  |  |  | |  | |  |  |  |  |
| **Cost per output** | | |  |  |  | |  | |  |  |  |  |
|  |  |  |  |  |  | |  | |  |  |  |  |
|  | Metric | | 2014 XOF | 2014 USD |  | |  | |  |  |  |  |
|  | Cost per household visited | | 64,608 | 131 |  | |  | |  |  |  |  |
|  | Cost per individual tested | | 9,050 | 18.3 |  | |  | |  |  |  |  |
|  | Cost per individual treated | | 332,524 | 673 |  | |  | |  |  |  |  |
|  |  |  |  |  |  | |  | |  |  |  |  |
